# Changes in the distribution of hydro-climatic extremes in a non-stationary framework

**DOI:** 10.1038/s41598-019-44603-7

**Published:** 2019-05-30

**Authors:** Taha B. M. J. Ouarda, Christian Charron

**Affiliations:** Canada Research Chair in Statistical Hydro-Climatology, INRS-ETE, 490 de la Couronne, Québec, QC G1K 9A9 Canada

**Keywords:** Projection and prediction, Hydrology, Atmospheric dynamics, Environmental impact

## Abstract

Hydro-climatic extremes are influenced by climate change and climate variability associated to large-scale oscillations. Non-stationary frequency models integrate trends and climate variability by introducing covariates in the distribution parameters. These models often assume that the distribution function and shape of the distribution do not change. However, these assumptions are rarely verified in practice. We propose here an approach based on L-moment ratio diagrams to analyze changes in the distribution function and shape parameter of hydro-climate extremes. We found that important changes occur in the distribution of annual maximum streamflow and extreme temperatures. Eventual relations between the shapes of the distributions of extremes and climate indices are also identified. We provide an example of a non-stationary frequency model applied to flood flows. Results show that a model with a shape parameter dependent on climate indices in combination with a scale parameter dependent on time improves significantly the goodness-of-fit.

## Introduction

An adequate knowledge of the characteristics of hydro-climatic extremes is essential for structure design and management. Extremes are often defined as the maximum values of a given variable over an interval, typically a period of one year. Frequency analysis allows to obtain an estimate of the probability of occurrence of specific extreme events. In extreme value theory, a theoretical probability distribution function is usually fit to the extreme variable time series and quantiles associated to return periods of interest are obtained. The selection of the probability function represents a crucial part of the frequency analysis. In classical frequency analysis, it is assumed that the extremes time series are independent and identically distributed. However, there is evidence that this assumption is not always met in reality. It is well known for instance that hydro-climatic extremes are influenced by large-scale low frequency climate oscillations^1–5^ and by climate change^6–8^. Non-stationary models have been developed to take into consideration climate variability and change^9,10^. In such models, distribution parameters are usually made conditional on covariates representing climate variability or the time. In the vast majority of non-stationary hydro-climate models, the location and/or scale parameters of the probability function are made dependent on covariates. However, shape parameters are usually assumed to be constant^9,11–15^.

Two assumptions are thus generally made in non-stationary frequency analysis: (1) the distribution function does not change and (2) the shape of the distribution function is constant. We propose here to verify these hypotheses using L-moment ratio diagrams. These diagrams have commonly been used for the selection of the appropriate probability distribution function to fit a given sample data^16^. They are used in the present study to evaluate the temporal evolution of the scale and shape of hydro-climatic extremes. They are also used to relate the changes in the scale and shape of the distribution to climate oscillation phenomena. The proposed approach is applied to long annual flood and annual extreme temperature time series from western Canada. The usefulness of introducing climate indices in the shape parameter of the probability distribution function in a non-stationary framework is also tested.

## Data

Two case studies are considered in the present work. The first case study deals with annual flood flows in the province of British Columbia, Canada. Maximum annual daily flows are extracted from the national Canadian HYDAT database for the station 08MG005 (50.336°N, 122.800°W). This station, with a record period from 1923 to 2014, is part of the Reference Hydrometric Basin Network (RHBN). The RHBN consists in a selection of stations with good quality data, long record periods, and basins that are relatively free of human influences such as regulation, urbanization and land-use change^17^. These stations are recommended for the use in detection, monitoring, and assessment of the impacts of climate change.

The second case study deals with extreme temperatures in the province of British Columbia, Canada. Maximum annual daily temperatures are extracted at two stations from the maximum observed daily temperatures during the period of May to September (MJJAS). Stations Quatsino (50.53°N, 127.65°W) and Fort St-James (54.46°N, 124.29°W) are selected because of their long record periods: from 1896 to 2010 and 1895 to 2010 respectively.

The following climate indices are considered: the Atlantic Multi-decadal Oscillation (AMO), the Southern Oscillation Index (SOI) as a measure of the El Nino/Southern Oscillation (ENSO) phenomenon, the Pacific Decadal Oscillation (PDO), the Pacific North American (PNA) pattern, the Atlantic Oscillation (NAO), and the Arctic Oscillation (AO) indices. Monthly time series were retrieved online form the National Oceanic and Atmospheric Administration (NOAA) web site, and are available from 1856 to present for AMO, 1900 to present for PDO, 1871 to 2011 for AO, 1821 to 2017 for NAO, 1851 to 2014 for PNA, and 1866 to present for SOI.

## Results

### Change in distribution function

**Figure 1:**
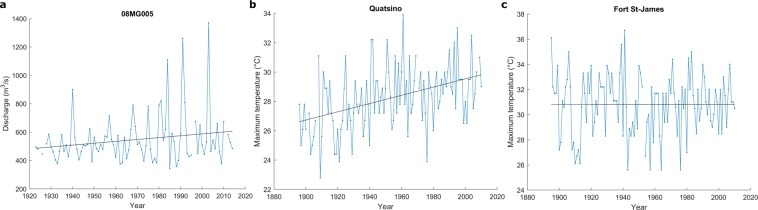
Time series and trends (dark line) at the studied stations.

**Figure 2:**
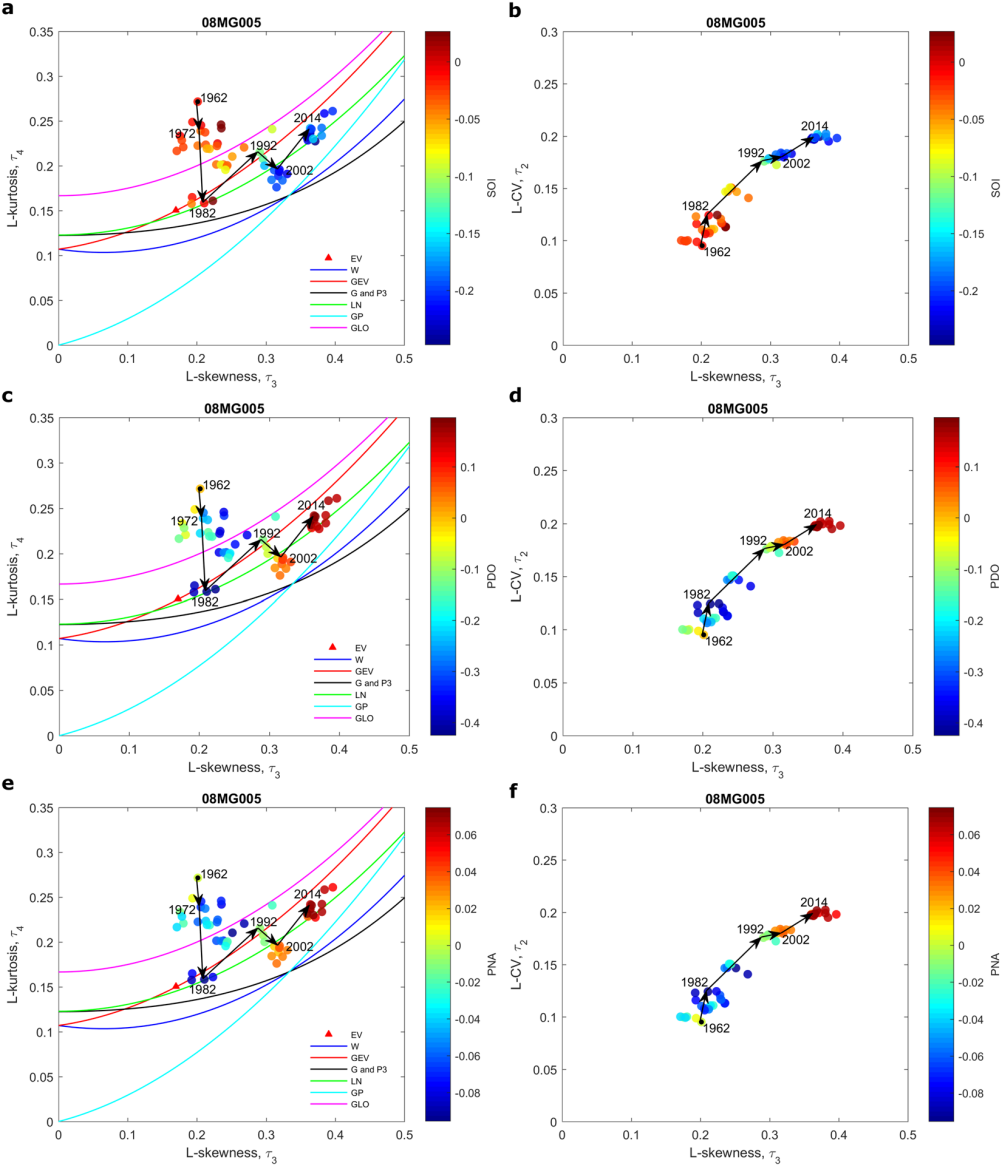
Scatter plots of 40-year samples on L-moment ratio diagrams of L-kurtosis vs. L-skewness (**a**,**c**,**e**) and L-CV vs. L-skewness (**b**,**d**,**f**) at the station 08MG005. The color of the point representing a 40-year sample corresponds to the mean value of SOI (**a**,**b**), PDO (**c**,**d**) or PNA (**e**,**f**) during the same period. Arrows denote temporal shifts in 40-year samples. A year denotes the last year of a given 40-year sample.

**Figure 3:**
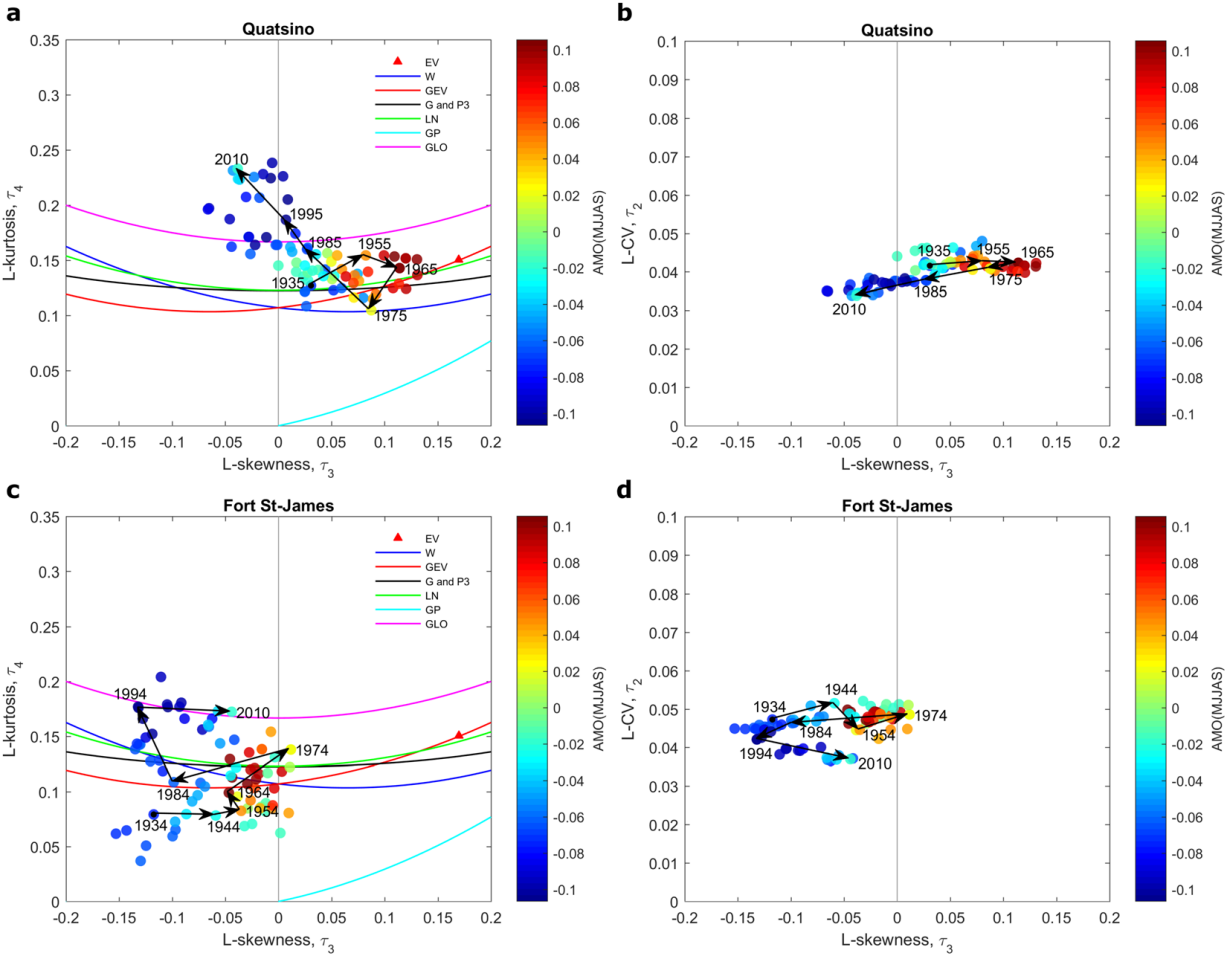
Scatter plots of 40-year samples on L-moment ratio diagrams of L-kurtosis vs. L-skewness (**a**,**c**) and L-CV vs. L-skewness (**b**,**d**) at the stations Quatsino and Fort St-James. The color of the point representing a 40-year sample corresponds to the mean value of AMO during the same period. Arrows denote temporal shifts in 40-year samples. A year denotes the last year of a given 40-year sample.

**Figure 4:**
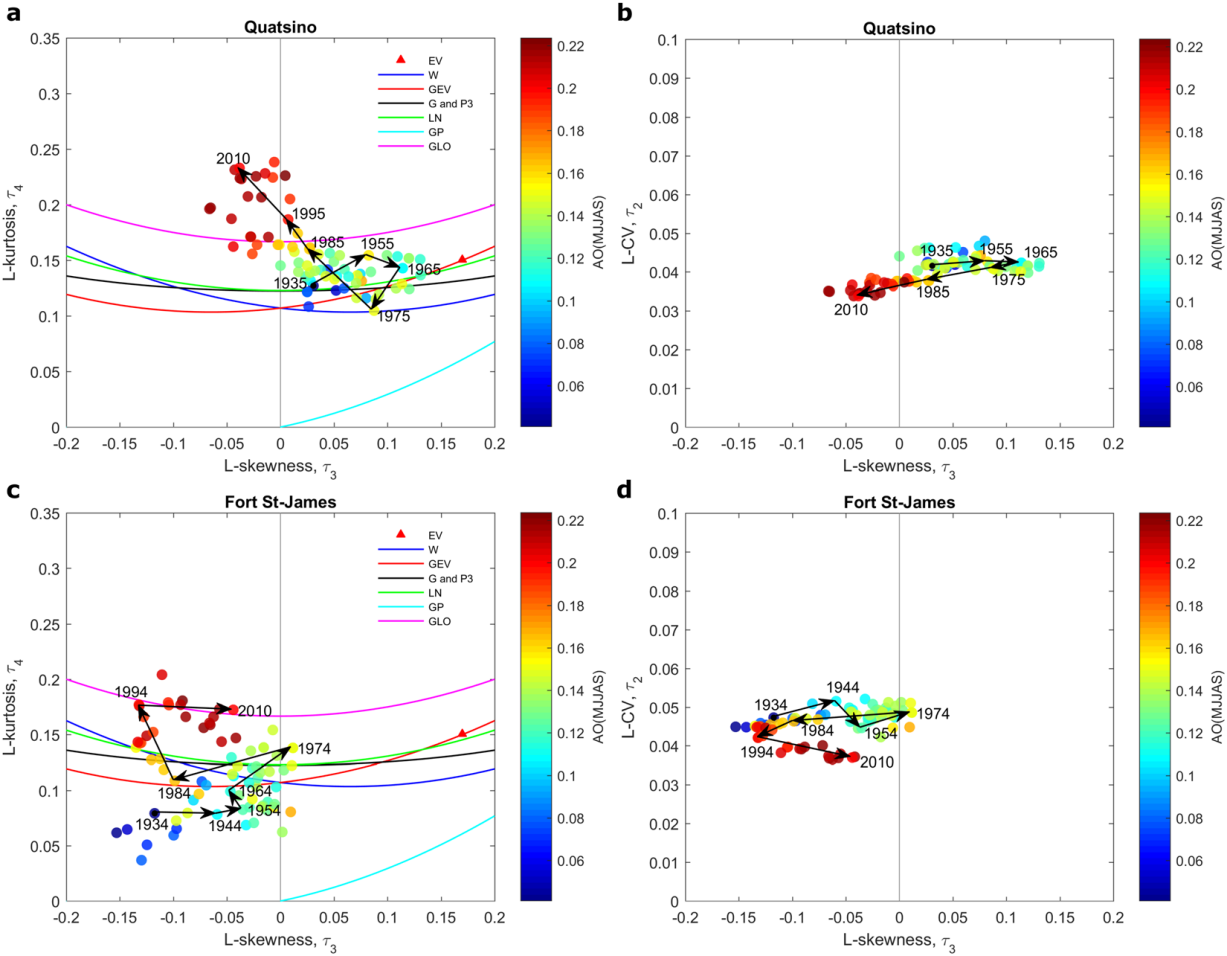
Scatter plots of 40-year samples on L-moment ratio diagrams of L-kurtosis vs. L-skewness (**a**,**c**) and L-CV vs. L-skewness (**b**,**d**) at the stations Quatsino and Fort St-James. The color of the point representing a 40-year sample corresponds to the mean value of AO during the same period. Arrows denote temporal shifts in 40-year samples. A year denotes the last year of a given 40-year sample.

Annual flood time series at station 08MG005 and maximum temperature time series at Quatsino and Fort St-James stations are illustrated in Fig. 1. The L-moment ratio diagrams for station 08MG005 are presented in Fig. 2 where 40-year samples are related to the climate indices SOI, PDO and PNA (See details in Methods). The L-moment ratio diagrams for extreme temperatures at Quatsino and Fort St-James stations are presented in Figs 3 and 4 respectively, where 40-year samples are related to the climate indices AMO and AO respectively. In Figs 2–4, 40-year samples for each 10 years from the beginning of the time series to the end, are connected with arrows. It is not possible to illustrate all 40-year samples in the diagrams as the figures will be too crowded. In these figures and in the present discussion, the year denotes the last year of the 40-year sample.

It can be observed in Figs 2–4 that the distribution that best fits the hydro-climatic data samples changes with time depending on the evolution of the location of the 40-year samples in the moment ratio diagrams. For instance, for the flood time series in station 08MG005, the distribution is clearly of the type GLO for the 40-year samples from 1962 to around 1982 to become approximately of the type LN or GEV for the 40-year samples from 1982 to 2014. The distributions associated to extreme temperature time series also change. In Figs 3 and 4 we can observe an increasing trend in the kurtosis and consequently a change of the distribution function that best fits the data. For Quatsino, the 40-year samples from 1935 to around 1965 can be of any of the distribution types LN, G or P3, or GEV, but the distributions of the 40-year samples from around 1985 to 2010 are of the type GLO. These results raise serious questions concerning the assumption commonly made in non-stationary hydro-climate modeling, that the parameters of the distribution may change but that the distribution remains the same. More effort needs to be devoted to this aspect in the future.

**Figure 5:**
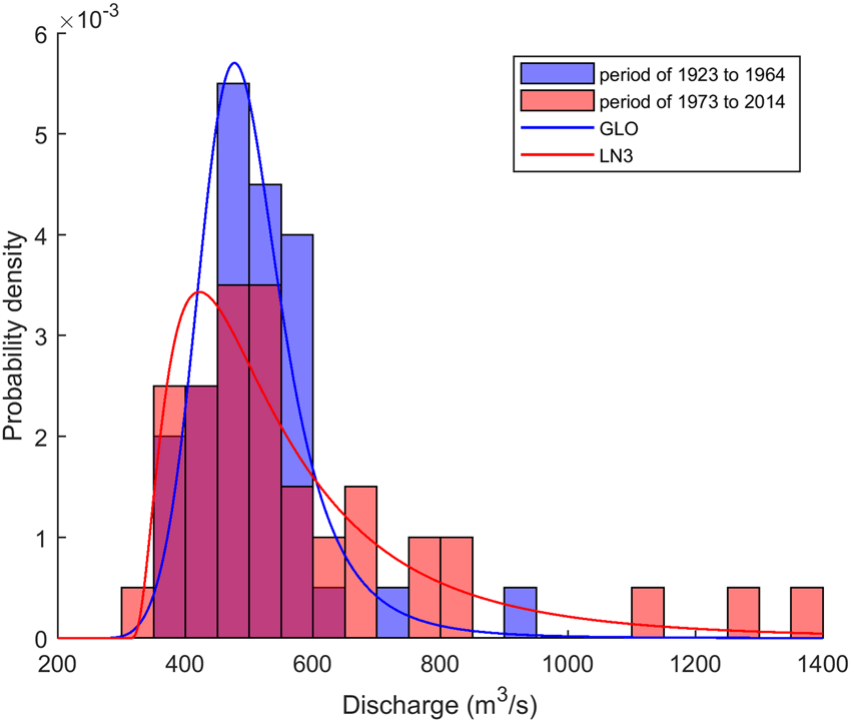
Frequency histograms for the 40-year sample corresponding to the period 1923–1964 and the 40-year sample corresponding to the period 1973–2014. The GLO is fitted to the sample of the period 1923–1964 and the LN3 is fitted to the sample of the period 1973–2014.

To illustrate the change in the shape of the distribution of the annual flood time series at station 08MG005, Fig. 5 presents the frequency histograms for the 40-year sample starting at the beginning of the time series (from 1923 to 1964) and the 40-year sample finishing at the end of the time series (from 1973 to 2014). Also, the probability functions GLO and LN3 are fitted to the sample of the period 1923–1964 and 1973–2014 respectively and are superimposed. These distribution functions are those that are most likely to fit each sample according to the L-kurtosis v. L-skewness diagram of Fig. 2. The shapes of the distributions for the period 1973–2014 compared to the period 1923–1964 show a clear decrease in the kurtosis, an increase toward right skewness and an increase in the variance (Fig. 5). These observations agree with the results obtained previously from the L-moment ratio diagrams in Fig. 2.

### Change in shape parameter

If a theoretical parent distribution is assumed for a given hydro-climatic extreme, the parameters of the distribution are also shown to change. For the flood time series, the GEV is a good model for the 40-year samples from 1982 to 2014. However, the skewness has also gradually increased during this period and thus also the shape parameter of the GEV. For instance, if the GEV is fitted to the flood data for the period 1914–1982, a value of *κ* = 0.09 is obtained. On the other hand, if it is fitted to the flood data for the period 1983–2014, a value of *κ* = 0.32 is obtained. Again, these results indicate that the commonly made assumption that the shape parameter does not change (see for instance El-Adlouni & Ouarda^18^) may need to be reviewed.

### Spatial coherence of non-stationary L-moment ratio diagrams

The Quatsino and Fort St-James temperature stations show a strong spatial coherence, i.e. a similar pattern is observed in the evolution of the 40-year samples with time in the L-moment ratio diagrams corresponding to the two temperature stations. Indeed, in both stations, a gradual increase in the kurtosis is observed in Figs 3 and 4. There is also a translation in the skewness of the 40-year samples toward zero from 1934 to 1974 followed by a translation toward more negative values from 1975 to 2010. This observation increases the confidence that the observed evolution in the L-moment ratio diagrams is not just random. This spatial coherence was observed for a large number of hydro-meteorological stations but space limitations prevent us from presenting these results.

### Climate variability: Influence of climate oscillations on the distribution shape

The relationship between climate oscillation indices and the shape of the distributions of hydro-climatic extremes is investigated here (see Figs 2–4). The whole year is considered for the computation of the averages of the climate indices SOI, PDO, PNA in Fig. 2. The reason is that the impacts of climate oscillations on flood flows range from the winter season, when climate indices have influence on snowmelt, to the autumn season, when they have influence on liquid precipitation. The summer season (MJJAS) corresponding to the period of extraction of the temperature extremes is considered for the calculation of the averages of the climatic indices AMO and AO in Figs 3 and 4 respectively. Note that these climate indices are selected based on previous studies that identified the oscillation modes which influence hydro-climatic variables in the Pacific region of Canada.

Figures 2–4 reveal possible links between the scale and shape of the distributions on one hand and climate indices on the other hand. In Fig. 2, skewness seems to be strongly influenced by climate indices. In all cases, there is a gradual shift in the color of the 40-year samples associated with the L-skewness, passing from one phase of the climate index to the opposite one. The variance seems also influenced by the climate index as there is a gradual shift in the L-CV (see the L-CV vs. L-skewness diagram) associated with the shift in the climate indices. Flood kurtosis seems not to be influenced by climate indices. The skewness and kurtosis of temperature extremes at Quatsino and Fort St-James stations seem also related to the climate index AMO (Fig. 3) and to the climate index AO (Fig. 4). In the case of extreme temperatures, the variance seems not to be influenced by climate indices.

### Non-stationary modeling with a conditional shape parameter

Non-stationary frequency analysis models with covariate-dependent shape parameters are developed and tested here (see Methods for model description and parameter estimation). The stationary GEV model and non-stationary GEV models are fitted to the time series of the station 08MG005. The GEV is selected because its curve in the L-kurtosis vs. L-skewness diagram seems to properly model the majority of the data samples displayed and is frequently used for the analysis of hydro-climatic extremes. Table 1 presents the stationary model and different non-stationary models fitted to the flood time series and the corresponding values of the Akaike information criterion (AIC) and the Bayesian information criterion (BIC). This station presents a variance that increases with time. This can be observed in the time series in Fig. 1 but also in the upward displacement of the curve in the L-CV vs. L-skewness diagrams in Fig. 2 where the L-CV doubles in value from 0.1 to 0.2. There is also an increasing trend in the mean value (location) according to Fig. 1a but attempts to include a temporal trend in the location parameter did not result in improved fits compared to a trend in the scale. The location parameter is thus considered constant for all non-stationary models as the trend in the variance is significantly more important than the trend in the location. Several non-stationary models are considered. In the first one, the scale parameter is considered conditional on time (i.e. ${\rm{GEV}}(x;\mu ,\sigma ={a}_{0}+{a}_{1}{\rm{Time}},\kappa )$). Since climate indices seem to have an impact on the shape of the distribution and consequently on the parameter *κ* of the GEV, the next three non-stationary models have a shape parameter *κ* that is conditional on the climate indices SOI, PDO and PNA respectively (e.g. ${\rm{GEV}}(x;\mu ,\sigma ,\kappa ={b}_{0}+{b}_{1}{\rm{SOI}})$). For the last three models, the time and a climate index are introduced jointly as covariates for the scale and shape parameters respectively (e.g. ${\rm{GEV}}(x;\mu ,\sigma ={a}_{0}+{a}_{1}{\rm{Time}},\kappa ={b}_{0}+{b}_{1}{\rm{SOI}})$).

**Table 1:** AIC and BIC Statistics for Nonstationary Models Applied to Flood flows at Station 08MG005.

Covariate	Model	AIC	BIC
—	${\rm{GEV}}(x;\mu ,\sigma ,\kappa )$	1098.38	1105.81
Time	${\rm{GEV}}(x;\mu ,\sigma ={a}_{0}+{a}_{1}{\rm{Time}},\kappa )$	1094.17	1104.08
SOI	${\rm{GEV}}(x;\mu ,\sigma ,\kappa ={b}_{0}+{b}_{1}{\rm{SOI}})$	1100.36	1110.27
PDO	${\rm{GEV}}(x;\mu ,\sigma ,\kappa ={b}_{0}+{b}_{1}{\rm{PDO}})$	1098.56	1108.47
PNA	${\rm{GEV}}(x;\mu ,\sigma ,\kappa ={b}_{0}+{b}_{1}{\rm{PNA}})$	1096.89	1106.80
Time + SOI	${\rm{GEV}}(x;\mu ,\sigma ={a}_{0}+{a}_{1}{\rm{Time}},\kappa ={b}_{0}+{b}_{1}{\rm{SOI}})$	1095.13	1107.52
Time + PDO	${\rm{GEV}}(x;\mu ,\sigma ={a}_{0}+{a}_{1}{\rm{Time}},\kappa ={b}_{0}+{b}_{1}{\rm{PDO}})$	1091.24	1103.62
Time + PNA	${\rm{GEV}}(x;\mu ,\sigma ={a}_{0}+{a}_{1}{\rm{Time}},\kappa ={b}_{0}+{b}_{1}{\rm{PNA}})$	**1090.08**	**1102.47**

Bold values denote best statistics.

The statistics AIC and BIC corresponding to the non-stationary models are compared to those of the stationary model. BIC penalizes more complex models than AIC for the long time series considered in this study. The inclusion of a trend in the scale parameter (variance) improves the goodness-of-fit. The inclusion of a climate index only in the shape parameter and thus neglecting the trend in the variance does not improve the goodness-of-fit except for PNA where the AIC is improved. However, results indicate that models that include a trend in the scale parameter along with a shape parameter that is conditional on PDO or PNA are the overall best models according to AIC and BIC. This means that when the temporal trend in the variance is modeled, the variability in the shape of the distribution can be explained by climate indices. The inclusion of the trend only in the scale parameter leads to a large improvement in the model goodness-of-fit according to the AIC and BIC criteria. For the considered case study, this model is even better than the one that also includes SOI in the shape parameter.

## Discussion

The analyses based on L-moment ratio diagrams demonstrate that the two commonly made assumptions in non-stationary frequency analysis of hydro-climatic extremes, i.e. that the distribution function and the shape of the distribution do not change, need sometimes to be reconsidered. In the case studies considered in this work, it was found that important changes in the distribution type and in the shape of the distribution can occur. Note that this finding was observed in a large number of other case studies that are not presented in this work because of space limitations. It was also found that there is a possible relationship between the shape of the distribution and climate indices for the case studies considered.

It is hence proposed that the common assumption in non-stationary models that the shape parameter is constant needs to be revised in some cases. Changes in the shape of the distributions are usually neglected in non-stationary models compared to changes in the location or scale. It is also commonly accepted that climate oscillations influence the location and scale of the distribution, but there is a lack of studies on their influence on the shape of the distribution. The addition of climate indices in the shape parameter of the non-stationary GEV model with a temporal trend on the scale parameter has resulted in improved goodness-of-fit in a flood flow case study used to illustrate this point.

The results presented here suggest that non-stationary models with covariate-dependent shape parameters can be of interest in hydro-climate extreme modeling. L-moment ratio diagrams can represent useful tools to analyse changes in the scale and shape of the time series distribution prior to carrying out a non-stationary frequency analysis. They can be useful to identify the appropriate distribution, to identify the parameters of the distribution that may depend on covariates, and to identify the potential covariates that have an influence on the scale and shape of the hydro-climate extreme distribution.

## Methods

### L-moment ratio diagrams

L-moments are defined as linear combinations of probability weighted moments. They were introduced by Hosking^19^ as alternatives to conventional moments over which they present important advantages: they are able to characterize a wider range of distributions, they are more robust to the presence of outliers in sample data and they are less subject to bias in the estimation^20^. The L-moment of order *r* is denoted by *λ*_*r*_. Ratios of L-moments are important values to characterize distributions. The L-moment ratio *τ*_2_ = *λ*_2_/*λ*_1_ (L-CV) is analogous to the conventional coefficient of variation while *τ*_3_ = *λ*_3_/*λ*_2_ and *τ*_4_ = *λ*_4_/*λ*_2_ (L-skewness and L-kurtosis) are analogous to the conventional skewness and kurtosis.

L-moment ratio diagrams represent a convenient way to characterize the probability distributions of hydro-climatic variables^21–23^. L-kurtosis is usually plotted against L-skewness in L-moment ratio diagrams. L-CV may also be plotted against L-skewness to represent two-parameter distributions. L-moment ratio diagrams have been frequently used to select the most appropriate theoretical distribution to fit a given data sample^24–27^. Candidate theoretical distributions are usually plotted on the L-moment ratio diagram of L-kurtosis vs. L-skewness where a probability function without a shape parameter will plot as a point, with one shape parameter as a curve, and with two shape parameters as an area. In this approach, all possible values of the L-skewness and L-kurtosis of a given distribution are represented in the diagram. To select the appropriate distribution for a sample data, the sample L-moments are computed and the sample’s location is plotted on the diagram as a point. The selection of an appropriate distribution can then be made based on the relative location of the sample to the candidate theoretical distribution functions displayed on the diagram.

### Study methodology

To analyse the temporal evolution of the scale and shape of distributions of hydro-climate extremes, moving time windows are used in conjunction with L-moment ratio diagrams. Successive windows of periods of 40 years are extracted from the extreme value time series with a time step of one year between windows. For each window, sample L-moment ratios are computed and the location of the 40-year sample is plotted on the moment ratio diagrams of L-kurtosis vs. L-skewness and L-CV vs. L-skewness. In this manner, the temporal evolution of the scale and shape of the distribution of the studied time series can be evaluated by the evolution of the 40-year samples in the diagrams. The theoretical distributions of interest are also displayed on the diagram of L-kurtosis vs. L-skewness. The distributions displayed in the L-moment ratio diagrams are the Gumbel (EV), Weibull (W), generalized Pareto (GP), gamma (G), lognormal (LN), generalized logistic (GLO), generalized extreme value (GEV) and Pearson type III (P3). The theoretical expressions relating L-kurtosis vs. L-skewness for the selected distributions can be given analytically or through polynomial approximations and are available in reference textbooks^20^.

The choice of a sample size of 40 years represents a compromise between the requirement to have enough windows in order to illustrate the temporal evolution of the time series characteristics in the L-moment ratio diagrams, and the need to have windows that are large enough for a reliable estimation of the sample L-moments. A sample size of 40 is sufficient to establish a dependable estimation of the L-moments^28^. It is common in environmental sciences to encounter samples of size 40 or smaller^29^.

Possible links between the shape of the distributions and certain low-frequency climate oscillation indices are also investigated here using L-moment ratio diagrams. For each 40-year sample of the hydro-climatic variable, the mean value of the climate index of interest is computed for the same period. A different color is assigned to each point in the L-moment ratio diagram depending on the mean magnitude of the corresponding climate index. The potential impact of the climate index can be evaluated by the pattern of colors displayed in the diagram. This method has limits since the immediate impact of climate indices on annual observations may not be well represented by the use of mean values for the climate indices over the window. For high frequency indices such as SOI, more than one cycle may be included in the 40-year window. On the other hand, for indices such as AMO, cycles can last for several decades and a 40-year sample may be more appropriate for the analysis. The mean value of the low frequency climate index contains then a stronger signal than for high frequency climate indices.

### Parameter estimation

The GEV distribution is commonly used to model hydro-climate extremes and it is the theoretical asymptotic distribution for annual maxima^30^. The probability function of the GEV is given by:

1 $$\mathrm{GEV}(x;\mu ,\sigma ,\kappa )=\begin{array}{ll}exp\{-{[1+\kappa (\frac{x-\mu }{\sigma })]}^{-1/\kappa }\} & \mathrm{if}\,\kappa \ne 0\\ exp[-exp(-\frac{x-\mu }{\sigma })] & \mathrm{if}\,\kappa =0\end{array}$$

where *μ*, *σ* > 0, and *κ* are the location, scale and shape parameters respectively, and $\mu -\sigma /\kappa  < x < \infty$ for *κ* > 0, $-\infty \le x\le \infty$ for *κ* = 0 and $-\infty \le x\le \mu -\sigma /\kappa$ for *κ* < 0.

For non-stationary models, distribution parameters are conditional on covariates. In the non-stationary framework, the parameters of the GEV can have different relations with covariates representing the time (temporal trend) and climate indices (teleconnections) for instance. The time covariate is defined by a series of integers incremented from 1 to the number of years in the time series.

Distribution parameters are estimated with the maximum likelihood method (ML). Given a probability density function *f* and a vector of parameters *θ*, the likelihood objective function for the sample $x=\{{x}_{1},\ldots ,{x}_{n}\}$ is given by:

2 $${L}_{n}=\prod _{t=1}^{n}f({x}_{t};\theta )$$

The estimator of *θ* is the vector $\hat{\theta }$ that maximizes *L*_*n*_ in Eq. (1). The Akaike information criterion (AIC) and the Bayesian information criterion (BIC) are commonly used for model selection. They are given by:

3 $${\rm{AIC}}=-\,2\,\mathrm{ln}({L}_{n})+2p,$$

4 $${\rm{BIC}}=-\,2\,\mathrm{ln}({L}_{n})+p\,\mathrm{ln}(n)$$

where *p* is the number of parameters of the model. AIC and BIC are indicators of the goodness-of-fit of the model to the data but account also for the parsimony with the parameters *p* and ln(*n*) which penalize more complex models with a larger number of parameters.

## Data Availability

Daily maximum temperature data were obtained from the online tool Historical Climate Data at the site http://climate.weather.gc.ca/. Maximum annual flows were retrieved from the Water Survey of Canada’s HYDAT database available at http://collaboration.cmc.ec.gc.ca/cmc/hydrometrics/www/. Climate indices were obtained from the NOAA’s Earth System Research Laboratory at http://www.esrl.noaa.gov/psd/data/climateindices/list/.
